# Identifying Autism from Neural Representations of Social Interactions: Neurocognitive Markers of Autism

**DOI:** 10.1371/journal.pone.0113879

**Published:** 2014-12-02

**Authors:** Marcel Adam Just, Vladimir L. Cherkassky, Augusto Buchweitz, Timothy A. Keller, Tom M. Mitchell

**Affiliations:** 1 Department of Psychology and Center for Cognitive Brain Imaging, Carnegie Mellon University, Pittsburgh, Pennsylvania, United States of America; 2 Machine Learning Department, Carnegie Mellon University, Pittsburgh, Pennsylvania, United States of America; 3 Brain Institute of Rio Grande do Sul (InsCer/RS), Pontifical Catholic University of Rio Grande do Sul, Porto Alegre, Rio Grande do Sul, Brazil; French National Centre for Scientific Research, France

## Abstract

Autism is a psychiatric/neurological condition in which alterations in social interaction (among other symptoms) are diagnosed by behavioral psychiatric methods. The main goal of this study was to determine how the neural representations and meanings of social concepts (such as *to insult*) are altered in autism. A second goal was to determine whether these alterations can serve as neurocognitive markers of autism. The approach is based on previous advances in fMRI analysis methods that permit (a) the identification of a concept, such as the thought of a physical object, from its fMRI pattern, and (b) the ability to assess the semantic content of a concept from its fMRI pattern. These factor analysis and machine learning methods were applied to the fMRI activation patterns of 17 adults with high-functioning autism and matched controls, scanned while thinking about 16 social interactions. One prominent neural representation factor that emerged (manifested mainly in posterior midline regions) was related to *self*-representation, but this factor was present only for the control participants, and was near-absent in the autism group. Moreover, machine learning algorithms classified individuals as autistic or control with 97% accuracy from their fMRI neurocognitive markers. The findings suggest that psychiatric alterations of thought can begin to be biologically understood by assessing the form and content of the altered thought’s underlying brain activation patterns.

## Introduction

Psychiatric disorders of thought are usually characterized and diagnosed on the basis of clinical assessment of an individual’s verbal and physical behavior. This is the conventional way to assess a thought disorder. However, recent advances in brain reading have made it possible to identify neurocognitive representations based on the underlying brain activation patterns assessed with fMRI [1]–[5]. These innovations have advanced from merely associating an activation pattern with a particular thought to decomposing the activation pattern into its neural and psychological components. For example, the activation pattern corresponding to the thought of a banana consists of components representing how one holds a banana (indicated in several premotor areas) and how one eats a banana (represented in eating-related areas). Another example is that the thought of an emotion such as sadness can be identified in terms of the neural representation of its valence, degree of arousal, and sociality [3]. Thus it has become possible to assess the content of a thought in neurotypical populations.

In our study, this approach was applied to characterize the *altered* neural representation of social concepts in autism, known to be disordered in terms of psychiatric diagnosis. If certain types of social concepts are altered in autism, it may be possible to (a) detect the alterations and possibly interpret them as diagnostic of autism; and (b) understand the biological and psychological nature of the alterations in terms of the underlying dimensions of neural representation; and (c) make use of the understanding to develop therapies that ameliorate the alteration. Furthermore, if the approach is successful with respect to autism, it may hold promise for application to other psychiatric disorders.

One of the largest challenges in autism research is to determine the relation between the psychological alterations in autism (assessed in behavioral and psychiatric studies) and the neural alterations (assessed in neuroscience and particularly brain imaging studies). Because the social alterations are often the most prominent ones in autism, fMRI studies of autism have investigated the relation between brain and behavior with respect to several different types of social processing. One of the earliest-studied social functions investigated with fMRI was face perception, during which it was found that the fusiform face area (a brain region associated with the processing of faces) activated abnormally in autism [6]. A second type of social task in which altered activation was found in autism was in Theory of Mind processing in which participants must understand the mental state of another individual (and in which there is altered activation in autism in the medial frontal and temporoparietal junction regions) [7].

A third type of autism alteration involved in social processing (and arguably the most central one) concerns the altered conception of *self* (see Uddin [8] for a review). The altered conception of *self* in autism is at the focus of the current study. Since its first description by Kanner [9], autism has always been prominently associated with a disruption of the social relation between *self* and others. In fact, the word *autism* stems from the Greek *autos* meaning *self.* Although *self* representation may have several types of components, such as visual self-recognition and perspective, the facet of *self* that seems most altered in autism is the relating of oneself socially to others. Individuals with autism exhibit atypical social behavior, manifested as disproportionate self-focus in social interaction with others. Hence the current study investigated a number of social (dyadic) interactions, using a neurosemantic paradigm in which participants are asked to think about a concept such as *to insult,* while their brain activation was assessed with fMRI.

Several fMRI studies of autism that have involved *self*-related cognition have found disruption of the brain activation in midline cortical structures (ventromedial prefrontal, middle and posterior cingulate), as summarized in a recent review [10]. One example is that in participants with autism there is a failure to reduce the activity in midline structures during the performance of a cognitive task [11], which has been attributed to a reduction of *self*-referential processing in the resting state in autism [12]. Another example of unusual *self*-related disruption in children with autism is the use of the pronoun *you* to refer to themselves, echoing the use of that pronoun by others to refer to the child, as first noted by Kanner [9]. This language behavior is ascribed to an errorful assessment of the relation between the *self* and another person. Consistent with Kanner’s observations, an fMRI study of pronoun processing in adult participants found diminished functional connectivity in autism between a frontal region (right anterior insula) and the precuneus (a posterior midline) region as well as altered activation levels in the precuneus [13]. Several other studies have found the precuneus to be involved in the representation of *self* [14], [15], [16], [17], [18]. Taken together, these types of findings indicate disruption of *self*-related processing in autism associated with the precuneus and frontal regions.

Findings of mean differences between autism and control groups in brain anatomy or brain activity have led more recently to classification studies in which participants are automatically (i.e. using an algorithmic statistical technique) classified as autistic or control based on such measures [19], [20], [21], [22]. Based on the structural grey matter anatomy measures, it was possible to classify the group membership with 85% accuracy [19]. With the voxel-based morphometry approach, the accuracy was 90% [20]. One study performed autism membership classification based on resting state connectivity data, producing an accuracy of 79% (and for the sub-group under 20 yrs, 91%) [21], whereas another study obtained an accuracy of 96% [22]. There is apparently something distinctive about the brain structure and brain activation in autism. However, neither of these approaches relates a brain property to a specific type of concept or thought that is altered in autism. The current study examines whether such classification is possible based on the neural representations of interpersonal social interactions, which might be expected to be altered in autism. In effect, the study seeks specific neurocognitive disruptions directly related to thought alterations and not simply biological markers of the thought disorder. We asked whether it is possible to distinguish autism from control participants based on their neural activation patterns during their consideration of various social interactions, examining whether the *self* components of social representations are altered in autism.

In addition to relating altered neural activation patterns to social concepts, the study attempted to determine what anatomical alterations in autism might be associated with the psychological alterations in the conception of *self*. One theory of autism relates the disorder’s behavioral and brain activation symptoms to altered frontal-posterior anatomical connectivity in the cortex, compromising the communication bandwidth between frontal and posterior areas [23]. The white matter tract that provides such connectivity between some of the main frontal and posterior midline regions involved in the representation of *self* is the cingulum bundle, whose structural properties can be measured noninvasively using magnetic resonance-based imaging of the diffusion of water molecules. An alteration in the representation of *self* could be due to the quality of this white matter tract. An a priori hypothesis was that the degree of alteration in the representation of *self* in individuals with autism would be related to the quality of their cingulum bundle. To examine this relation, diffusion images of this tract were obtained, in addition to the fMRI activation evoked by thoughts of various social interactions.

Another hypothesis was that the degree of alteration in the representation of *self* in individuals with autism would be related to behavioral measures of various social abilities, such as face processing and Theory of Mind (c.f. [12]). To test this hypothesis, appropriate neuropsychological measures were acquired for participants with autism.

Autism is rightly considered to be a heterogeneous disorder, with suggestions made that it be referred to as “the autisms” [24]. There are anecdotal comments that every person with autism is autistic in their own way. Although autism is undoubtedly heterogeneous, a striking finding in brain reading studies of neurotypical people is the high degree of commonality (homogeneity) of neural representations of concepts across individuals. A classifier trained to identify the thoughts associated with physical objects like a banana from the neural activation patterns of a group of participants can then identify, with reasonable accuracy, the thoughts of a new participant whose data were not included in the training [2]. This activation commonality probably arises because of the commonalities in the structure, function, and experience of human brains as they process information related to physical objects. But how would a psychiatric or neurological disorder affect the commonality among the members of the affected population, particularly in a domain of thought that is altered in the disorder? Given the apparent heterogeneity of autism, should there thus be less commonality among people with autism than among people without autism when they are thinking about social concepts? That is, if autism entails altered conceptions of social interactions, are the alterations heterogeneous across people with autism or is there a commonality? New machine learning methods allow a comparison of the commonality within the autism and the control groups.

The central issue remains whether it is possible to identify a participant as autistic, not just on the basis of a fortuitous statistical relation, but on the basis of some fundamental alteration of the brain activity that underpins particular types of thought that are among the defining characteristic of the disorder.

Below we first apply factor analysis to reduce the dimensionality of the brain activation evoked by the various social interactions. Then we perform classification of the multivoxel patterns that correspond to particular social interactions in order to identify the interaction and to distinguish the neural patterns of the two groups. The advantages of the approach are that it 1. focuses on the representations of social interactions, which are likely to be altered in autism and which like other concepts, are neurally represented by multiple voxels in multiple regions, and 2. is capable of detecting group differences in the activation patterns of multiple voxels in multiple regions.

## Materials and Methods

The study acquired fMRI-measured brain activation patterns of 17 young adults diagnosed with high-functioning autism and 17 age and IQ-matched control participants as they thought about the referent of 8 social interaction verbs (*compliment, insult, adore, hate, hug, kick, encourage, humiliate*), considered from two perspectives (either the agent of the action or the recipient), for a total of 16 social interaction items. There were 6 presentations of such 16-item blocks.

### Ethics statement

The study protocol was approved by the University of Pittsburgh and Carnegie Mellon University Institutional Review Boards. All participants gave their informed written consent.

### Participants

The participants’ demographic information is shown in Table 1. The diagnosis of autism was established using the *Autism Diagnostic Observation Schedule* (ADOS; [25]), the *Autism Diagnostic Interview-Revised* (ADI-R; [26]) using DSM IV criteria and confirmed by expert clinical opinion. All participants were required to be in good medical health. Seven of the autism participants took medications on the day of the scan (six of these taking selective serotonin re-uptake inhibitors, three taking ADHD medications, two taking blood pressure medications, and three taking one of prostate enlargement, hypothyroidism, or allergy medication). Potential participants with autism were excluded if they had an identifiable cause for their autism such as fragile-X syndrome, tuberous sclerosis, or fetal cytomegalovirus infection or were found to have evidence of prematurity, birth asphyxia, head injury, or a seizure disorder. Exclusions were based on neurologic history and examination, physical examination, and chromosomal analysis or metabolic testing, if indicated. The control participants were community volunteers and were group-matched to the participants with autism on age, gender, race, and all three IQ scores, Verbal (VIQ), Performance (PIQ), and Full-scale (FSIQ) as determined by administration of the Wechsler Abbreviated Scales of Intelligence (WASI; [27]). Potential control participants were screened by questionnaire, telephone, face-to-face interview, and observation during initial testing and were excluded if they had a current or past history of prematurity, psychiatric and neurologic disorders, birth injury, developmental delay, school problems, acquired brain injury, learning disabilities, or medical disorders with implications for the central nervous system. Exclusionary criteria also included a history in first degree relatives of autism, developmental cognitive disorder, affective disorder, anxiety disorder, schizophrenia, obsessive compulsive disorder, or other neurologic or psychiatric disorder thought to have a genetic component. One of the control participants took allergy and asthma medication and another participant took an antibiotic on the day of the scan.

**Table 1:** Age, IQ, handedness, and gender of the participants.

	Autism Mean (Range, SD)	Control Mean (Range, SD)	t(32)	p
Age (years)	25.6 (16–38, 6.7)	23.4 (17–36, 5.2)	1.06	0.30
VIQ	113.8 (87–132, 14.7)	111.4 (94–134, 9.5)	0.57	0.57
PIQ	112.6 (92–131, 11.8)	113.8 (104–135, 9.3)	0.34	0.74
FSIQ	114.9 (92–132, 13.4)	114.2 (100–139, 9.5)	0.18	0.86
Handedness	13 Right: 4 Left	13 Right: 4 Left		
Gender	15 Male: 2 Female	17 Male: 0 Female		

Note: VIQ = Verbal IQ; PIQ = Performance IQ; FSIQ = Full-Scale IQ.

Handedness was determined with the Lateral Dominance Examination from the Halstead-Reitan Neuropsychological Test Battery [28]. Thirteen members of each group were right-handed; two of the autism and none of the control participants were female.

Prior to in-scanner testing, each participant was familiarized with the task, and used an MRI simulator scanner to acclimate themselves with the scanner environment. The 34 included participants were tested in two epochs. In the first epoch, 9 participants with autism and 9 controls were scanned using a Siemens Allegra scanner, with 21 additional participants excluded from the analysis (as described below). Because the yield was low (18/39) in the first epoch largely due to excessive head motion, the pre-scanning training to reduce head motion was substantially enhanced in the second epoch. The yield for the second epoch (16/20) was greatly improved. In the second epoch, 8 autism and 8 control participants were scanned using a Siemens Verio scanner with 4 additional participants excluded (using the same criteria).

The data from the 25 excluded participants (12 with autism and 13 controls) had been affected by either excessive (above 3.5 mm) head motion (6 with autism and 3 controls) or lack of attention to the stimulus in a substantial number of trials (6 with autism and 10 controls). Participants in such studies comment that occasionally their mind wanders when processing some items, and we have previously found such inattention to be characterized by an abnormal occipital activation time course. Consequently, participants in whom the abnormality (measured as a low correlation with a typical occipital activation time course) was frequent (occurring in more than 70% of the items) were excluded. (Calculations are shown in File S1).

### Image acquisition

Functional images were acquired on a Siemens Allegra 3.0T or a Siemens Verio 3.0T MRI scanner (Siemens, Erlangen, Germany) using the same gradient echo EPI sequence with TR = 1000 ms, TE = 30 ms and a 60° flip angle. Seventeen 5-mm thick oblique-axial slices were imaged with a gap of 1 mm between slices. The acquisition matrix was 64×64 with 3.125×3.125×5 mm voxels. High angular resolution diffusion images (HARDI) were acquired using a diffusion-weighted, single-shot, spin-echo, EPI sequence (TR = 5300 ms) and processed using FSL tools and diffusion toolkit software [29]. (See File S1 for details).

### Stimuli and paradigm

The stimulus set of eight verbs referring to interpersonal actions (*compliment, insult, adore, hate, hug, kick, encourage, humiliate*) was presented one at a time, with instructions to think about the nature of the interaction from either the perspective of the agent (e.g., the participant insulting someone else) in a dyadic situation, or from the perspective of the recipient (e.g., being insulted by someone else), for a total of 16 different social interactions. Each block of 16 interactions (8 verbs × 2 perspectives) was presented 6 times. In each block, the two perspectives were presented separately and always in the same order for a given participant (and balanced across participants), while the 8 verbs within each perspective were presented in different random orders. There was a 10 s rest interval between blocks and also between perspectives within a block. The mean interval between the two consecutive presentations of the same verb was 66 s, and the maximum interval was 115 s.

Each stimulus verb was presented on the screen for 3 s, followed by a 4 s rest period, during which the participants were instructed to fixate on an X displayed in the center of the screen. There were four additional presentations of a fixation condition X, 24 s each, distributed across the session to provide a baseline measure of activation.

Participants were asked to think about the most salient properties of the interaction that the verbs described, for example, whether the action is intentional or not, the reaction it may evoke, and the context in which it occurs, to encourage consideration of multiple attributes of the dyadic social interaction. Participants were asked to think of the same attributes each time they saw a given verb. To encourage the consideration of a consistent set of attributes, prior to the scanning session participants were asked to write down the attributes of each verb in each mode/role. However, there was no attempt to induce consistency across participants.

### Neuropsychological tests

To assess the social processing abilities of the autism participants, the Benton Facial Recognition Test [30], WMSIII Faces II [31], and Reading the Mind in the Eyes [32] were administered.

### fMRI processing

The fMRI data were preprocessed with SPM2 [33]. For each participant, functional images (about 15,000 voxels) linked to every instance of the 16 social interaction terms were computed and served as input data for the following analyses (see File S1 for further details of fMRI data preprocessing).

### Factor analyses

To assess the neural representation of social interactions, a two-level, exploratory factor analysis (FA), as described in previous research [2], was applied separately for each group. This dimension reduction approach aims to identify the relatively sparse set of cortical regions and voxels whose neural activity varies reliably across the set of stimulus items, while representing the relevant neural activity for each participant in a way that allows multiple participants’ data to be aligned and compared. The choice of parameter values in the procedure was determined by search and convergence in several previous studies. For example, the total number of voxels ultimately involved in the analysis, 135, is small, relative to the entire brain volume. However, our previous studies showed that increasing this number failed to substantially improve the classification accuracy and at some point the accuracy begins to decrease with additional voxels [2]. Several of the arbitrary-looking procedures below are the result of optimizations performed in several previous studies.

The details of the factor analysis procedures (starting with the initial selection of 135 voxels per participant and ending with the uncovering of 4 major factors per group, together with the associated brain locations), are reported in the File S1.

**Figure 1:**
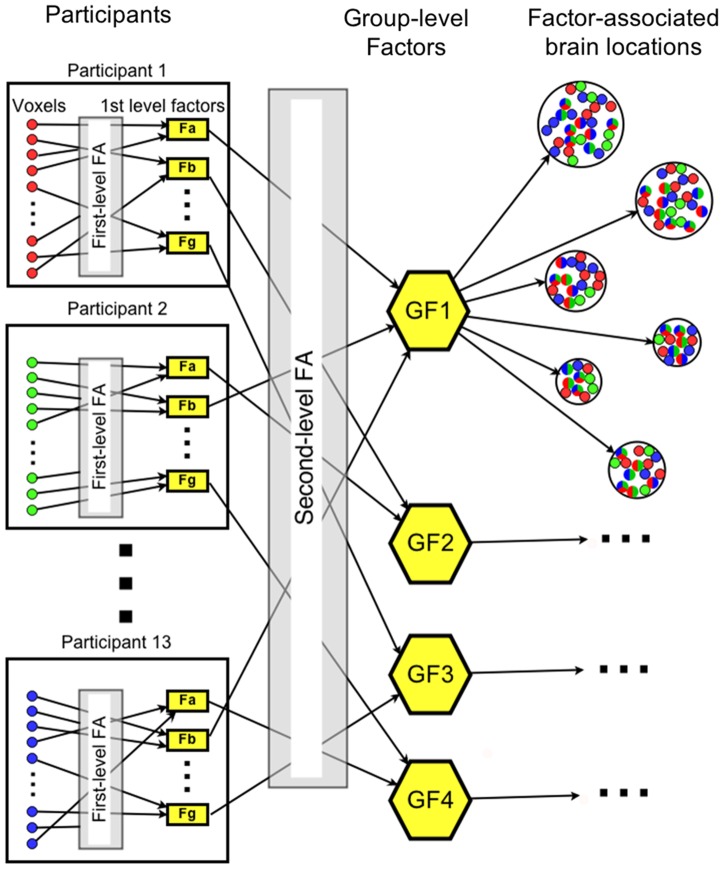
Schematic diagram of the two-level exploratory factor analysis procedure. The first level factor analyses are performed separately for participants 1–13. In these analyses, the activation levels of 135 voxels (marked as red, green, and blue circles for the 3 participants) distributed throughout the brain are expressed via 7 factors (Fa-Fg), and some (but not all) of the voxels are linked to these factors. The second, group-level FA in turn expresses the 13×7 first-level factors in terms of 4 group factors (GF1–GF4). For each of these factors, the originating voxels are spatially clustered. A cluster of such voxels (characterized as a sphere) contains voxels that were initially selected from many (typically all) of the participants. The six largest spheres per factor were treated as the factor-associated brain locations.

The FA procedure for a group of participants is illustrated in Figure 1. The 135 most stable voxels distributed across 5 brain areas were algorithmically selected for each participant. The first-level FA was performed separately for each participant, resulting in 7 first level factors (Fa-Fg, Figure 1). (The number of first-level factors was fixed at 7, which was the modal number of factors for all participants based on the Kaiser criterion). These factors were characterized by their vector of scores for the 16 items and their associations with specific subsets of the initially selected 135 voxels. The goal of the first-level FA was to find the participant-specific distributed brain networks involved in the representation of social interactions.

The second, group-level FA then attempted to find the components of these networks that were common across participants within each group. The group-level factors (GF1–GF4, Figure 1) were also characterized by their vector of scores for the 16 items and their associations with specific subsets of the first-level factors, and, through these associations, to subsets of originally selected voxels. The spatially contiguous clusters of these voxels (factor-associated brain locations in Figure 1) defined the brain locations of the neural representation components corresponding to the group factors. The number of factors in the group-level FA was limited to 4, beyond which they were not easily interpretable, and the locations were limited to the 6 largest clusters (characterized as spheres) per factor.

The only outcome of the factor analysis that was used in the subsequent machine learning (described below) was the set of locations (centers and radii) of the factor-related spheres. The features (voxels) used in the classification came exclusively from these factor-based spheres (but were subject to additional criteria).

### Machine learning analyses

Gaussian Naïve Bayes (GNB) classifiers with factor-based features were used to classify participants’ group membership and separately, to identify the 16 social interactions (see File S1 for the details of machine learning computations).

#### 1. Group membership classification

This classification was performed separately for each participant, training the classifier on the remaining participants, and deriving the features from the locations of the semantic factors that emerged from the factor analyses. Specifically, the features were derived from the union of the 3 semantic factor locations from the autism group’s analysis with the 3 semantic factor locations from the control group’s analysis. Thirty-six spherical volumes were created (from 2 groups, 3 factors, 6 spheres per factor; each sphere was defined by voxels with the highest loadings for the factor). Each sphere was characterized by the activation levels of its representative voxels across the 16 social interactions derived from the participants’ responses. The 16 activation levels of the 5 most stable voxels in a sphere were averaged and then converted to z-scores. (The stability of a voxel was defined as the similarity (correlation) of its pattern of activation responses to the set of 16 interactions across the 6 presentation blocks.) The same procedure was applied to all participants, including the test participant, resulting in a set of features consisting of 576 values (36 spheres x 16 stimulus items) for each participant. Only 115 of these features were used, namely those with the largest absolute value difference between the group means in the training set (any number of features between 80 and 290 resulted in the same classification accuracy of 0.97). The machine learning procedure trained the classifier on these data from 33 of the participants (each labeled as autistic or control), and then it attempted to classify the remaining participant. In each of these 34 iterations of classification, the training and test data were kept completely separate, including 34 separate factor analyses.

#### 2. Classification of individual social interactions

The second type of classification attempted to identify to which of the 16 social interactions a given brain image corresponded. The latter classification was performed both within participants (re-iteratively dividing the participant’s data into training and test sets) and across participants in a group (training the classifier on data from 16 participants and identifying the social interactions in the data of the 17th, left-out participant). (see File S1 for details).

## Results

### Overview of main findings

- The neural activation patterns associated with social interaction concepts such as *hug* and *adore* in individuals with high-functioning autism lack a subcomponent of neural activity in posterior cingulate/precuneus, which is strongly evident in control participants. This finding emanated from a factor analysis of the activation patterns of 135 automatically selected voxels (volume elements, each 59 mm^3^) from each participant distributed throughout their brain. For reasons discussed below, we interpret this subcomponent of neural activity as associated with *self*-related cognition.
- The individuals in the autism and control groups can be identified as such automatically with high specificity and sensitivity by a machine learning classification of the neural activity associated with these social concepts. This result was obtained when a machine-learning classifier based on the factor analyses and trained on the data of all but one left-out participant was able to correctly predict whether or not that participant had autism in 33 of 34 (97%) of the cases.
- An individual’s neural representation of a particular social interaction (out of the 16) can be reliably identified at far above chance level by a machine learning classifier that has been trained on the neural activity from the *same individual* in an independent set of trials, indicating a systematic relation between brain activity and the thought about a particular social interaction.
- An individual’s neural representation of a particular social interaction can similarly be reliably identified at far above chance level by a machine learning classifier that has been trained on the neural activity of *other members* of their own group, indicating a commonality of neural representations across individuals. This outcome attests to the similarity of the alteration across people with autism.
- The degree of alteration of the neural representation of *self* in an individual with autism is correlated with the quality of the brain connective anatomy (cingulum bundle) joining regions associated with the representation of *self* (frontal and posterior midline brain areas). The degree of alteration is also correlated with behavior (face processing ability as measured with the Benton Facial Recognition Test [30] and other tests), thus providing a multi-tiered account linking the neural activity, brain anatomy, and behavior associated with an individual autistic participant’s thoughts about a particular social interaction.

This summary of results provides an overview but the details follow below.

### Factor analysis results

**Figure 2:**
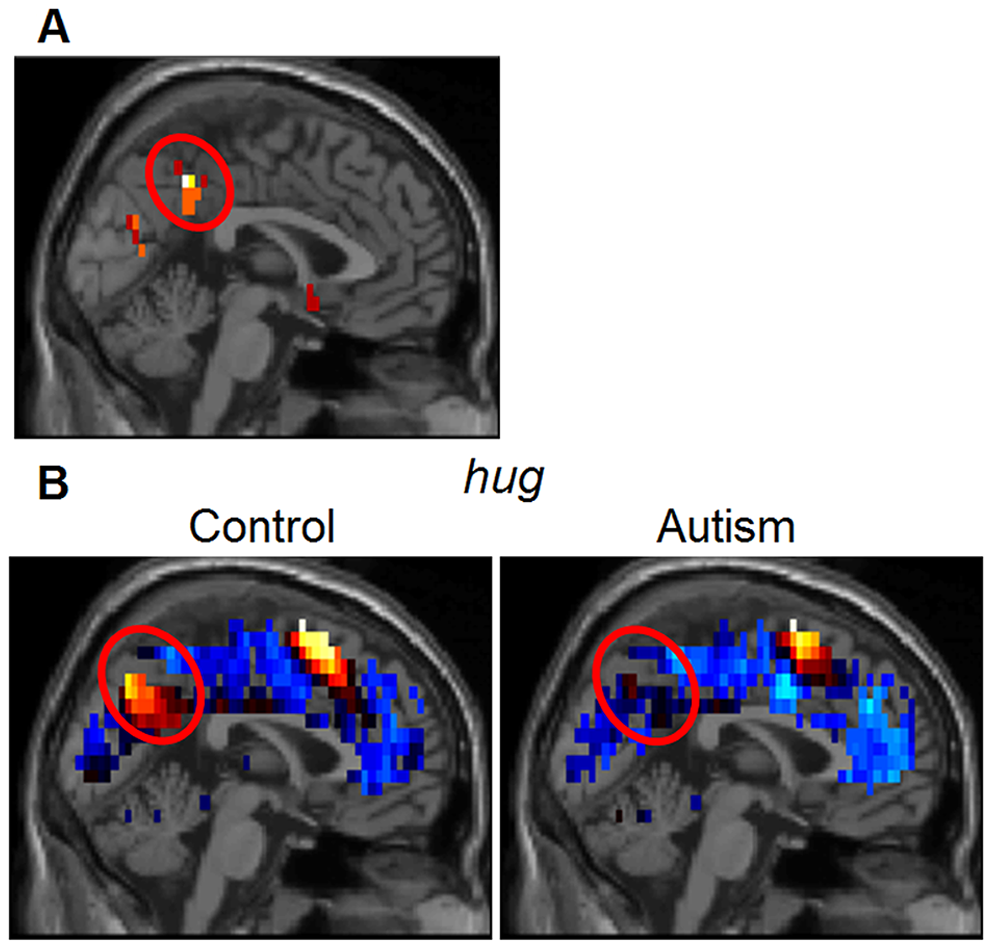
Posterior midline *self* factor location. A. Location of the voxels (circled) derived from the factor analysis of the Control Group that defined the posterior cingulate/precuneus sphere of this group’s *self* factor. Voxels in this cluster (with MNI x-coordinates extending from 0 to −9) are shown projected on the mid-sagittal plane. (The coordinates and radii of all 6 spheres associated with this factor are shown in Table S1 in File S1). B. Mean activation in midline brain structures for the verb *hug* (averaged over agent and recipient roles) for the two groups, differing in posterior cingulate/precuneus. The verb *hug* was chosen for illustration here because of the salience of hugging as a social interaction in autism, where enveloping pressure is sometimes desired but without physical contact between oneself with another person, as in Temple Grandin’s squeeze machine [40]. The depiction of the activation in this slice for all of the other verbs was very similar to *hug*, for both groups.

The main group difference was the presence of a factor in the control group’s activation with strong representation in the posterior cingulate/precuneus area, a factor that was absent in the participants with autism. We interpret this factor in the control group as being involved in *self*-related cognition, for two reasons. First, one of the main brain locations associated with this factor, the superior midline areas of posterior cingulate and precuneus, has been activated in many previous fMRI studies when a thinking task involved consideration of the *self*, and furthermore, several studies have reported that in autism this component of neural activation is disrupted [13], [34]. (The voxel locations most associated with the factor in this area are shown in Figure 2A. The complete set of 6 cortical locations for this factor and the other factors are shown in Table S1 in File S1.).

The second facet of the results that is consistent with the interpretation of the *self*-related factor is the ordering of the 16 social interactions by their factor scores for this factor, particularly the items at the two extremes of the 16-item ordered list. The two items with the highest factor scores were *hate* in the agent role and *humiliate* in the recipient role (followed by *hate*/recipient and *insult* in both roles). The two lowest-ranking interactions were *kick* in the recipient role and *kick* in the agent role. By contrast, the autism group had no factor that ordered the interactions similarly nor which had a substantial posterior cingulate factor location, indicating a diminished degree of representation of the *self* in autism in the context of these social interactions.

Figure 2B shows the difference between the two groups for the verb *hug* in the agent role, indicating the relative absence of activation in posterior cingulate/precuneus in autism compared to the control group. Although it was previously known that there is sometimes abnormally low activation in autism in the posterior midline areas, the new results here indicate much more precisely how this region’s role is modulated by the degree of *self*-involvement in the control group, and hence what is altered in the autism group.

Regardless of this factor’s precise interpretation, the coding of the social interactions by this factor and the others makes it possible to identify whether an individual participant belongs to the autism or control group, and furthermore to identify which social interaction he or she is thinking about at a given time, as described below.

In the autism group’s activation, the comparably ranked factor appears to instead encode how physical the actions were. We base this interpretation on the main brain regions associated with the factor, particularly L precentral (a motor-related area) and L postcentral (a somatosensory area). The four interactions with the highest scores from this factor are *kick* in both roles, *hug* in recipient role, and *encourage* in the agent role, all of which entail a physical action). The four lowest-ranked interactions were *hate* and *insult* in both roles.

The remaining three factors were similar between the two groups. We interpret these three factors as coding for the positive or negative valence of the social interaction, the accessibility/familiarity of the interaction, and the length of the verb name, factors which we describe in turn.

The social valence factor assigned high factor scores to socially positive interactions (e.g. *adore, compliment*) and low scores for negative interactions (e.g. *humiliate, hate*). The valence factors of the two groups were very similar, assigning highly correlated (r = .96) factor scores to the 16 interactions. The brain locations for this factor included caudate and putamen for both groups.

The factor interpreted as accessibility or familiarity produced factor scores for the 16 interactions that were highly correlated (r = .89) between the two groups. Furthermore, the brain locations associated with this factor, very similar for the two groups, included regions that are part of the default mode network, particularly middle cingulate, R angular gyrus, and R superior medial frontal. Our interpretation of this factor is based in part on the assumption that the more accessible the social interaction was, the more resources were left over to activate the default network. According to this interpretation, activation of the default mode network here is not an indication that these regions are semantically encoding familiarity, but that their pattern of activation is a byproduct of the ease or difficulty of semantic access. For example, for the control group, interactions with the highest accessibility/familiarity scores were *compliment* and *hug* whereas *insult* and *adore* had the lowest scores.

The word length factor was extremely similar for the two groups both in terms of the brain locations (strongly associated with L and R Occipital pole for both groups) and factor scores for the 16 interactions (their correlation was r = .99) which were also highly correlated with the number of letters in the verb name (r = .98 for both groups), with *compliment* and *hug* anchoring the factor for both groups. The 4 factors together accounted for 41.8% of the variation for the autism group and 43.0% for the control group, with most of the factors accounting for similar amounts (9.3–10.6%), except for the word length factor which accounted for slightly more (13.3% for autism; 13.2% for controls).

In summary, the factor analyses indicate a major group difference, namely that the autism group lacked a *self* factor and instead had a factor corresponding to the verbs’ impersonal semantic (abstract-physical) properties.

### Classification of participants as autistic or control

A machine learning classifier (GNB) that was based on the union of the two groups’ factor analyses (minus the participant being classified) was able to identify each participant as autistic or control with very high accuracy (33 of 34 or 97% of participants correctly classified), misclassifying one participant with autism as a control. The features of this classifier were derived from 3 factors from the autism group’s factor analysis (*physical-abstract*, *social valence*, and *accessibility*) and 3 factors from the control group (*self*, *social valence,* and *accessibility*), excluding the word length factor, which was very similar for the two groups. The pattern of brain activation levels for the 16 interactions in the set of 36 locations associated with the factors reliably distinguished the two groups. This outcome confirms the postulated differential neurocognitive representations of social interactions for the two groups, and indicates the substantial diagnostic potential of this approach.

In summary, the differences in the ways that people with autism in this sample neurally represent interpersonal interactions can be used by a classifier to identify a person as having autism or not, with high accuracy.

### Classification of social interactions

It was possible to identify which of the 16 social interaction items a participant was thinking about, based on the neural representation of the 4 factors that emerged from each group’s factor analysis. A GNB classifier was trained on an independent subset (4 of the 6 presentation blocks) of each participant’s own data and then tested on their remaining subset (the mean of the other 2 presentations blocks). Each of the 16 items was characterized by its activation level in 24 spheres (6 spheres for each of the 4 factors) for that participant group. The resulting mean rank accuracies (hereafter, accuracies) for classifying the 16 items were reliably (*p*<.001) above chance level (0.56) for all participants (with mean accuracies of 0.71 for the autism group and 0.68 for the control group). The successful classification of individual social interactions indicates that the factor analysis captured important components of their neurosemantic representation.

Another striking finding was the ability to identify which of the 16 social interactions a participant with autism was thinking about by training the classifier exclusively on the factor analysis-guided activation data of the other autistic participants (again using the same 4 factors from the autism group). This classification produced a mean rank accuracy of 0.82 in the autism group, with all 17 autism participants’ social interaction classification accuracies falling reliably (*p*<.001) above chance level (0.72). (The higher mean classification accuracies across participants than within participants may be due to the larger amount of training data in the former case). That the representation of a social interaction in a participant with autism could be decoded by training a classifier solely on data from other people with autism indicates substantial commonality of the neurosemantic alterations across people with autism. Despite the well-known heterogeneity of autism, the alteration of the neural representation of these social concepts is apparently similar across the autism participants.

Similarly, there was commonality across the control participants, where the corresponding classification produced a mean accuracy of 0.77, with 16 of 17 control participants’ classification accuracies falling reliably (*p*<.001) above chance level.

### Relation to anatomical connectivity and behavioral measures of social processing

Diffusion imaging was used to determine whether the altered representation of *self* in autism is related to the quality of the cingulum bundle, the anatomical tract that connects the frontal and posterior regions involved in the representation of *self.* The measure of each autism participant’s cingulum tract quality was the mean density across all voxels in the tract (computed from MNI-space density maps representing the number of fibers passing through each voxel in the tract [35]. The measure of an autism participant’s rudimentary degree of representation of the *self* was the mean stability of their 3 most stable voxels in the main location (posterior cingulate/precuneus) of the control group’s *self* factor. The L cingulum tract density measure (corrected for participants’ age) was positively correlated (*r* = .50, p<0.05) with the rudimentary degree of representation of *self.* (The correlation for the R cingulum tract was lower and not reliable, but in the same direction, *r* = .17). This result indicates that better anatomical connectivity in a participant with autism between posterior and anterior midline areas (both of which have been involved in *self*-related activity in previous studies) was associated with stronger rudiments of a *self* factor.

**Figure 3:**
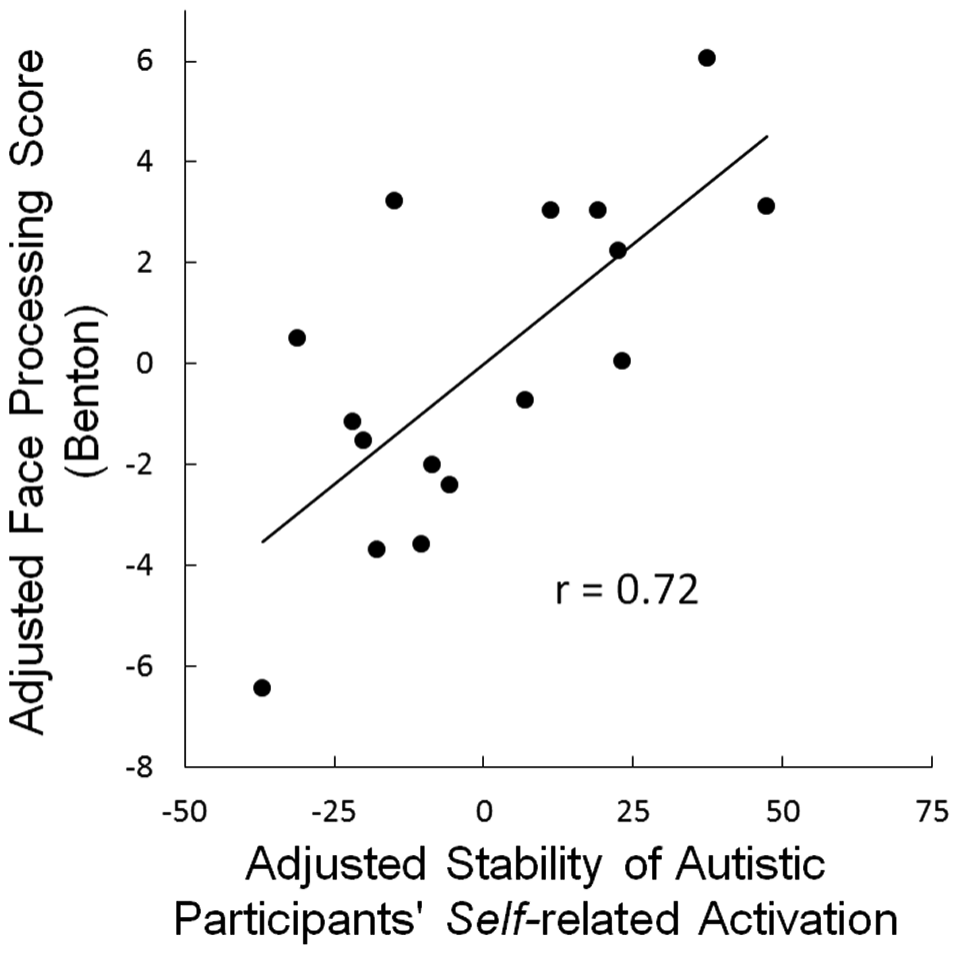
Degree of alteration of *self*-related activation in autism (estimated by its stability in posterior cingulate/precuneus) and its relation to social processing ability measured by the Benton Facial Recognition Test [30]. Both measures were adjusted for participants’ age and full scale IQ. One participant with autism did not have a Benton Test score.

The strength of these *self* rudiments (corrected for participants’ age and full scale IQ) was also positively correlated with each of the behavioral measures of social processing: the Benton Facial Recognition Test score (*r* = 0.72, *p*<.05) [30], as shown in Figure 3; WMS III Faces II (r = .69, *p*<.05); and Reading the Mind in the Eyes (r = .78, *p*<.005). In addition, the correlation of the *self* rudiments with the ADOS social total was -.21 n.s. (the negative correlation is in the expected direction). The indicated p-values are Bonferroni-corrected for the 4 comparisons.

### Conventional general linear model (GLM) analyses

**Figure 4:**
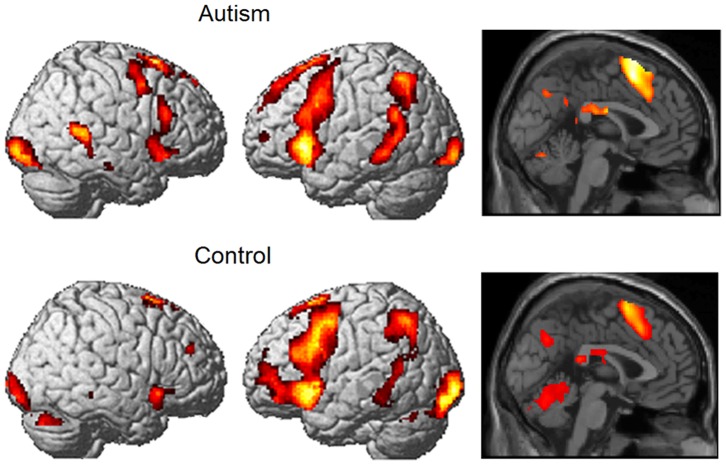
Social Interactions-Fixation contrasts for the two groups. The uncorrected *p*-threshold is 0.001 and the extent threshold is 5 voxels for both groups.

GLM contrasts revealed none of the main findings of the multivariate approach. A between-group SPM contrast (Autism-Control) (all social interactions – fixation), explored with an uncorrected threshold of p = 0.001 and extent threshold of 5 voxels showed essentially no group differences (the autism group’s activation was higher in two small clusters (6 and 7 voxels) located in right superior and middle frontal gyri). The within-group contrasts (all social interactions-fixation) showed activation in similar areas for the two groups, including left inferior frontal gyrus, left superior temporal gyrus, superior frontal, left middle frontal and middle temporal, left inferior parietal areas, and bilateral occipital pole, as shown in Figure 4. The group with autism additionally activated right inferior frontal gyrus, middle frontal and middle temporal areas.

## Discussion

The main finding provides a plausible biological basis for the psychological phenomenon of altered conceptions of social interaction in autism. The factor analyses indicate the autism group lacked a *self* factor and instead had a factor corresponding to the verbs’ impersonal semantic (abstract-physical) properties. The participants with autism may have viewed the social interactions referred to by the verbs as though they themselves were a spectator (like an “anthropologist on Mars,” as described by Temple Grandin, referring to how a person with autism might view complex social interactions without self-involvement [36]). This new approach to characterizing the nature of thought alterations provides a new meaning to the concept of *biomarker*, which is usually thought of as a biological marker of a biological state. Here we see a set of brain activation patterns constituting a biological marker of a set of *altered cognitive states* corresponding to conceptions of social interactions. The biological alteration in the brain activity corresponding to the alteration of the thought pattern can be considered a neurocognitive marker of autism. This overview provides a guide to the discussion section but the detail and substantiation follow below.

The neurosemantic group difference is much weaker in non-social semantic domains. A small pilot study of 6 adults with autism and 6 controls examined whether the two groups differed to a similar extent in their neurosemantic representations of 10 tools and 10 dwellings [37], two semantic domains that might be expected to be represented rather similarly in autism and in controls. Approximately similar machine learning methods produced substantially less accurate group membership classification, identifying group membership correctly for no more than 7 of the 12 participants (chance level accuracy would be 6 of 12), failing to find a statistically reliable group difference in the neural representation of concrete objects.

Although the current findings with only 34 participants must be treated with caution, they help close the loop relating *brain activation patterns* and *brain anatomy* in autism to *thought* and *behavior*, suggesting a causal path. The evidence we have reported above shows that (a) the group differences in activation patterns in response to social interactions are sufficient for automated identification of autism; (b) the main distinction of the autism activation pattern was the near absence of systematic activation in a midline posterior cingulate/precuneus region associated with the representation of *self,* indicating a lack of psychological self-involvement in these social representation; (c) furthermore, in individuals with autism, the residual strength (stability) of the *self*-related activation rudiments in this brain area was correlated with the density of fibers connecting that area to a frontal region, which is also involved in *self*-related cognition; and the residual strength of the *self*-related activation rudiments was also correlated with behavioral measures of the autism participants’ social processing ability. The correlation with anatomy may be of particular interest because recent genomic research has uncovered several genetic alterations (copy number variants and single nucleotide variants) sometimes found in autism that are capable of altering axonal development and maintenance during early neurodevelopment, potentially leading to altered connectivity in the affected axonal tracts [24], [38], [39]. Thus, alterations in frontal-posterior brain connectivity may underlie the altered social behavior and brain activation observed in autism.

One extension of this approach may be to the study of alterations in autism of thoughts of emotions. A recent brain-reading study has applied this method to identifying which of 18 emotions a neurotypical participant was experiencing, finding (a) high identifiability of emotions; (b) high commonality across participants; and (c) a set of 3 neural factors underlying the emotions (valence, intensity, sociality) [3]. These findings suggest that it should be possible to assess alterations in emotion representations in autism and other disorders using the current approach.

### Study limitations

Despite the very high sensitivity and specificity of the approach (33/34 or 97% of participants classified correctly), the study has clear limitations. First, the current paradigm, requiring significant cooperation during thoughts about social interactions, would be difficult to apply to participants with lower-functioning autism. Second, it is not yet known whether this type of classification can differentiate autism from other special populations, such as those with other developmental and neurological disorders. Furthermore, it would be desirable to develop a neurosemantic screening battery that contains a variety of items capable of evoking altered representations in a number of psychiatric disorders, along with a classifier that accurately identifies the disorder of individual participants. Each disorder could then be identified or diagnosed on the basis of its own characteristic alterations of thought. Because of the many co-morbidities among psychiatric disorders, one might expect classification of some individuals into more than one category. Fortunately, these limitations have the potential of being overcome through further research efforts.

### Factor analysis implications

There are several implications of the various factor analyses, most generally indicating that it is feasible to determine the underlying dimensions of neural representation of social concepts. Despite the fact that a concept evokes activation in many different locations, it is possible to apply dimension reduction techniques like factor analysis or principal components analysis to converge on a small set of factors or dimensions that can account for much of the systematicity of the activation. In the case of the 16 social interactions examined in the current study, the three dominant dimensions were the *self*-related factor for the control group or the physicality factor for the autism group, as well as the positive/negative valence of the interaction and the accessibility/familiarity of the interaction. These are proposed to be the underlying dimensions of the neural representations of social interactions. The names we have given each of the factors reflect our interpretations of them, which in turn are based on the each factor’s associated brain locations and its ordering of the 16 interactions. Because social interactions seem such an intrinsic concern of the human mind, it seems plausible that there exist a core set of dimensions for thinking about them.

Regardless of the interpretability of the recovered underlying dimensions in a neural representation space, the mere presence of such factors, common over participants, suggests the possibility of there being a small number (say 50–200) of fundamental neural dimensions of representation that underlie all concepts. In effect, these dimensions would constitute a basis set, from which the representation of any concept could be constructed. It would remain to be seen whether any such basis set would be exclusively biologically given or whether there could also be experience-based dimensions that are part of the basis set. The idea of a basis set of this type is highly speculative, but as brain imaging research progresses it will become increasingly possible to assess.

One of the assumptions of this study was that the thought alterations in autism are underpinned by a perturbation of some fundamental dimension of neural representation, which the results suggest may be the *self*-related dimension. More generally, it is possible that other psychiatric disorders may be characterized by a perturbation of a particular neural dimension of representation. For example, it is possible that paranoia may be characterized by a perturbation (overactivity) of a threat-detection dimension of representation. Perhaps psychiatric disorders that are currently characterized by verbal descriptions of altered behavior and thoughts may someday be characterized by altered neural dimensions of representation that can be localized to particular sets of brain regions that represent a particular property.

The finding of a commonality of representation among the participants with autism reveals a facet of autism that stands in contrast to the well-known heterogeneity of the disorder. Although people with autism surely differ enormously among themselves, they must nevertheless have something in common. Almost everyone with autism has some alteration in social processing, but the form of the altered behavior can differ among people, for a variety of reasons, from people developing idiosyncratic coping strategies to people having different mixtures of gene alterations. But there has to be something at the core of the disorder that may be a defining characteristic. Many studies have characterized the behavior or the brain activation in autism as being altered, but often without specifying the nature of the alteration in terms that speak to its commonality across people with autism. The current results provide a possible core property, the neural representation of social interactions, that is altered similarly across participants with autism, namely in that the representation of *self* is largely absent.

This finding supports theories of autism that postulated altered representation of *self* in autism [10], [12]. The correlation between the alteration of *self*-representation and the density of the cingulum bundle (which anatomically connects frontal and posterior regions involved in the representation of *self* is also consistent with the theory of frontal-posterior underconnectivity in autism [23].

The contribution of the machine learning is its demonstration that the factors and their locations are capable of accurately discriminating between participants with and without autism. The outcome of the factor analysis itself indicates that the dimensionality of the fMRI data can be reduced, but it does not provide evidence that the resulting dimensions are meaningful or useful. The machine learning provides this demonstration, showing that one of the emerging dimensions, namely *self*-representation, characterizes autism sufficiently well to enable accurate classification. Not for the first time, the multivariate machine learning analysis showed greater sensitivity to systematic activation differences than did univariate GLM contrasts.

One potential application of the current approach is to provide a biological measure of altered social processing in autism that can augment conventional structured-interview measures, as well as neuroanatomical and brain activity biomarkers of autism. A second potential application is to provide a precise enough characterization of altered social representations in autism to allow the design of targeted therapies and neuropsychiatric diagnostic procedures. Furthermore, both applications of this approach may be feasible with other psychiatric disorders which entail a systematic alteration of particular concepts, such as delusions. But the most far-reaching scientific significance is that psychiatric alterations of thought can begin to be biologically understood in light of their direct psychological consequences using brain imaging techniques in combination with machine learning analyses.

## Supporting Information

File S1 **Supporting Methods, Tables S1 and S2, and References.**
(DOC)
