# Supplementary material for: Identifying Autism from Neural Representations of Social Interactions: Neurocognitive Markers of Autism
Source: PLoS One. 2014 Dec 2;9(12):e113879. doi: 10.1371/journal.pone.0113879 (PMC4251975; doi:10.1371/journal.pone.0113879)
Supplement: File S1 — Supporting Methods, Tables S1 and S2, and References. (DOC) [file pone.0113879.s001.doc]

Supporting Information for:

Identifying Autism from Neural Representations of Social Interactions:

Neurocognitive Markers of Autism

Marcel Adam Just, Vladimir L. Cherkassky, Augusto Buchweitz, Timothy A. Keller,

Tom M. Mitchell.

Supporting Methods

**fMRI data preprocessing**

The fMRI data were preprocessed with SPM2. The data were corrected for slice timing, motion, linear trend, high-pass filtered using a 190s cutoff, and transformed to MNI space (without re-sizing voxels or spatial smoothing). The percent signal change (PSC) relative to the fixation condition was computed at each voxel for each item presentation. Following procedures in a similar study [1], the mean PSC of the four images acquired within a 4s window that started 4s after stimulus onset (to account for the delay in hemodynamic response) provided the main input measure for the analyses. The PSC data for each item were further normalized to have a mean of zero and variance one. An intersection of spatially normalized images from all participants was used as a common mask for analysis, i.e. the analysis was restricted to voxels (about 16,000) that were present in the images of all participants.

**Task inattention assessment based on visual activation**

We have previously found that participant reports of inattention during the processing of particular items is correlated with an aberrant time course of activation in primary visual areas, and that the data from participants whose inattention occurs on a large percentage of the items produces poor classification. Consequently, the time course of the activation in occipital pole ROIs (spheres with MNI centers (-18 -98 -6) and (18 -98 -6) and 6 mm radii) was assessed for each item for each participant by comparing it to a linearly detrended template time course. The template was derived from the mean activation of 5 occipital pole voxels averaged over all items of the participant with the most accurately classified individual items (social interactions).

The detrended time course for each item for each participant was computed using the mean of the 5 voxels per ROI with the highest overall correlation with the template time course (computed over all items). An item was considered unattended if its time course correlation with the template was less than 0.5 (based on pilot studies). A participant’s entire dataset was excluded if the percentage of unattended items was above 70% or 75%, depending on whether their in-plane (xy) head motion range was less than 1.5 mm.

**Factor analyses methods.**

**1. Initial selection of 135 stable voxels per participant.** Each voxel was first assigned a *stability* score using the data from the 6 presentations of each of the 16 stimuli, assessing how similarly (stably) the voxel responded across the set of 16 items in each of the presentations. Each voxel was first assigned a 6 x 16 matrix, where the entry at row i, column j, is the fMRI activation level (percent signal change from rest) of this voxel during the ith presentation of the jth stimulus. The stability score for this voxel was then computed as the average pairwise correlation over all pairs of rows in this matrix. This measure assigns higher scores to voxels that exhibit consistent (across different presentations) variation in activity across the 16 stimuli. For each participant, 135 voxels were then selected to include major regions as follows: the 30 most stable voxels from each of the frontal lobe plus anterior cingulate cortex, the temporal lobe, the parietal lobe plus posterior cingulate cortex, and the occipital lobe, as well as 15 voxels from subcortical areas (excluding cerebellum). These regions were defined by the Automated Anatomical Labeling (AAL) atlas [2].

**2. Factor analysis algorithm.** A Matlab implementation of a principal factor analysis algorithm (equivalent to the SAS v. 9.2 ([http://www.sas.com](http://www.sas.com/)) factor analysis procedure) including varimax rotation was used. The factor scores *f* for individual stimulus items were obtained by solving the equation below. In this equation, let *y_vi_^(p)^* be the observed fMRI activity for participant *p* responding to stimulus *i* at voxel *v*. The first level factor analysis provides a set of *N* (=7) factors, each characterized by their factor scores *f_ni_^(p)^* for participant *p*, and their factor loadings *l_vn_^(p)^*, such that:

N

*y_vi_^(p)^*  **= ∑** *l_vn_^(p)^  f_ni_^(p)^*

n =1

A second level of factor analysis, based on the output of the first-level analyses, was performed separately for each group to obtain 4 group-level factors underlying the interpersonal verb representations. Just as the first level analysis expresses the *y_vi_^(p)^* values in terms of factors *f* and their loadings *l*, the second level analysis expresses the first level factor scores *f_ni_^(p)^* in terms of participant-independent factor scores and loadings that relate them to the first level factor scores. Thus, given a set of second level factor scores and loadings, and the first level factor scores and loadings, we can reconstruct the observed *y_vi_^(p)^*. Importantly, the group-level factor scores and loadings taken together with the corresponding brain locations (see below) provide the basis for comparing the representations for the two groups of participants.

In addition to the two-level factor analyses, single-level FAs (one per group) which expressed the activation levels of voxels from all group participants directly in terms of group factors, were also performed and they produced factor structures that were somewhat similar to the two-level analyses but the factors were less interpretable.

**3. Identifying cluster-based spheres.** Each factor from the second level factor analysis was then associated with the set of six spherical brain regions in which the factor’s fMRI activity was most strongly expressed. This association was based on the loadings from both levels of the FA. In the first (participant) level FA, we observed that the majority of the 135 voxels had high loadings (positive or negative) on just one of the 7 first-level factors. Consequently, those of each participant’s 135 voxels that had some loading of at least .4 were mapped to the factor for which it had the highest loading.

At the second (group) level, those of the 119 first-level factors (17 participants x 7 factors) that had some loading of at least .4 (absolute value) on one of the group factors were mapped to the factor for which it had the highest loading. This procedure resulted in each group factor being mapped to a set of the voxels (contained in the union of the initially selected 135 voxels per participant). For each factor, its associated voxels were then used to derive 6 spherical regions defined by spatial clusters of these voxels. To specify the clusters for each factor, the factor’s voxels were plotted in MNI space and the spatial clusters of these voxels were identified. For the 6 clusters with the greatest number of voxels, corresponding spheres were defined. Supporting Table S1 presents the center of mass and the radius (defined as mean distance of all cluster voxels from the center; if this distance was less than 6mm, the cluster radius was set to 6mm) of each of the resulting clusters and spheres, for each of the 4 second level factors.

The factors of each group, serving as a basis of the representation of social interactions, were then interpreted in terms of the factor locations (spheres) and the ordering of the 16 social interactions by their factor scores, and compared for the two groups.

**4. Using factors to specify features for machine learning.** The factor scores and spheres served as the basis (features) for the identification of group membership and identification of social interactions using machine learning algorithms. In the machine learning applications, group-level FAs were performed on the data from only 16 of the 17 participants in the group, excluding the participant whose data is about to be classified. Factor scores and locations were identified for each analysis. The factors in each 16-participant FA were mapped to the corresponding full-group factors (by means of the highest correlation), identifying the subset of factors to be used in the machine learning classification, as described below. In all of the machine learning analyses, not only were the training data set and test data set kept completely separate, but also the factors were never derived from the data of the participant that was being classified.

**Machine learning computations**

Classifiers were trained to identify mental states associated with thinking about the social interactions, using the evoked patterns of functional activity (mean PSC). Classifiers were functions f of the form: f: mean_PSC → Yj, j={1, …,m}, where Yj were one of the binary categories (group membership) or the 16 social interactions, and where mean_PSC was a vector of mean PSC voxel activations associated with a set of factor-determined spherical volumes, as described below. To evaluate classification performance, data were divided into training and test sets. A classifier was built from the training set, and classification performance was evaluated on the left-out test set, to ensure unbiased estimation of the classification error. Our previous exploration indicated that several classifiers produced comparable results. In the interest of simplicity, we report results from one feature selection method and one classifier.

**1. Classification***.* We used the Gaussian Naïve Bayes (GNB) pooled variance classifier [3]. It is a generative classifier that models the joint distribution of a class Y (e.g. autism and control) and attributes (spheres), and assumes the attributes X1,…,Xn are conditionally independent given Y. The classification rule is:

n

*Y* ← arg max P(Y = y_j_) ∏ *P*(*X* *_i_* | *Y = y _j_*), *j* = 1,2,…,m,

*y_j i_*

where m is the number of classes (either 2 or 16) and n is the number of spheres.

The classes were approximately equally frequent. Classification results were evaluated using k-fold cross-validation, where one example per class was left out for each fold. In a two- and 16-class classification problem, chance level is 0.5. Rank accuracy was used to evaluate 16-class classification.

**2. Preliminary feature selection for classification***.* The features used for classification were the activation levels within a set of spheres (volumes) associated with the factors, 6 spheres per factor. Each sphere was characterized by the properties of the activation profile of 5 of its contained voxels, selected as described below. Using the factor spheres as features (as opposed to using voxels) served two purposes: first, it provided a way to map from the factor analysis results to the machine learning feature selection procedures; second, it allowed for a small amount of individual differences in the precise location of voxels associated with a given factor. The feature selection and cross-validation procedure for the *group membership classification* is described in detail in the main paper.

**3. Classification of individual social interactions***.* To identify which of the 16 social interactions a participant was thinking about, all group factors (4 factors per group) were used.

*Within participant classification.* For cross-validation over the 6 presentations, the data were iteratively partitioned into all 15 possible subsets of 4 presentations used for training and the mean of the remaining two presentations used for testing. (This protocol of averaging over two presentations for testing purposes was used because the data from a single presentation was found to be too noisy to allow accurate neurosemantic classification in several previous studies).

The factors obtained from the 16-participant FA (excluding the participant being classified) were used to specify 6 spheres per factor (similarly to the group membership classification). A maximum of 5 voxels with the highest cross-presentation stability scores were selected from each sphere (to characterize the sphere), based on the voxel stability computed over the 4 training presentations. The same voxels were selected to represent the sphere in the training and test presentations. The activation levels of the selected voxels were averaged to obtain the 16 activation levels for each sphere.

**Classification across participants within a group**. For the analysis across participants within each group, one test participant at a time was excluded from the training set, and a 16-participant FA was used to specify 6 spheres per factor. A maximum of 5 voxels were selected from each sphere; for both the training and test participants, these were the voxels with the highest cross-presentation stability scores. The 16-item activation levels of selected voxels were averaged across the 6 presentations and the selected voxels within a sphere. There were 16 such sets of data used for training the classifier (from 16 participants) and a set of data from the test participant.

**High angular resolution diffusion-weighted imaging (HARDI)**

Diffusion data were collected using a diffusion-weighted, single-shot, spin-echo, echo-planar imaging (EPI) sequence with TR = 5300 ms, TE = 95 ms, bandwidth = 1860 Hz/Voxel, FOV = 200 mm, and matrix size = 128 x 128. Thirty-six 3-mm thick slices were imaged (no slice gap) with no diffusion-weighting (*b* = 0 s/mm^2^, 8 repetitions equally spaced throughout the acquisition), and with diffusion-weighting gradients applied in 61 orthogonal directions (*b* = 1000 s/mm^2^). The diffusion data were preprocessed (corrected for motion and eddy currents) using FSL tools (<http://www.fmrib.ox.ac.uk/fsl/fdt/index.html>). The acquired gradient vector tables were rotated to match the motion-corrected volumes, and orientation density functions were reconstructed with Diffusion Toolkit (DTK) software [4] (http://www.trackvis.org/dtk), using the spherical harmonic version of the Q-ball method [5]. Whole-brain deterministic tractography was performed with DTK software using default parameters. Segmentation of the left and right cingulum bundle from each participant’s whole-brain tractography followed previously described protocols [6] and using TrackVis software [4]. The mean density of the fibers (adjusted for brain size via transformation to MNI space) was then calculated for each participant’s left cingulum bundle.


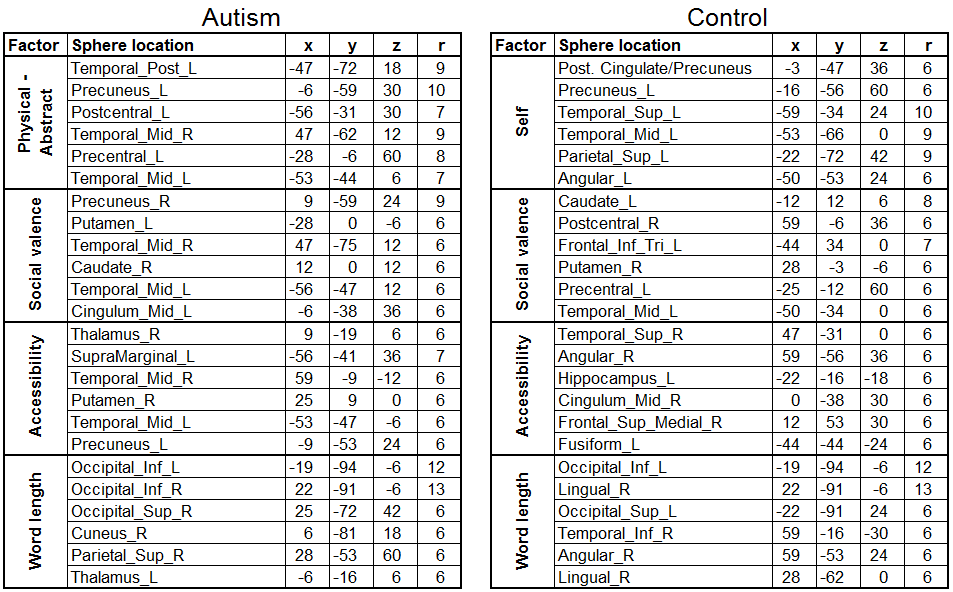


**Supporting Table S1.**

Locations and radii of factor spheres for the autism and control groups. Anatomical labels, taken from the AAL atlas, describe the location of the main coverage of the sphere. The variation accounted for by autism group factors was: Physical-Abstract: 9.5%; Social valence: 9.7%; Accessibility: 9.3%; Word length: 13.3%, and by the control group factors, Self: 10.6%; Social valence: 9.3%; Accessibility: 9.9%; Word length: 13.2%.

|  | Autism | | | |
| --- | --- | --- | --- | --- |
|  | Physical-Abstract | Social Valence | Accessibility | Word Length |
| Highest scores | **kick** | **adore** | **encourage** | compliment |
|  | **encourage** | adore | **hug** | humiliate |
|  | hug | **compliment** | hate | **encourage** |
|  | **…** | **…** | **…** | **…** |
| Lowest scores | **hate** | kick | kick | hate |
|  | **insult** | humiliate | **kick** | hug |
|  | insult | **hate** | **insult** | **hug** |
|  |  |  |  |  |
|  | Control | | | |
|  | Self | Social Valence | Accessibility | Word Length |
| Highest scores | **hate** | **adore** | compliment | **compliment** |
|  | humiliate | adore | **hug** | humiliate |
|  | hate | **compliment** | **humiliate** | **encourage** |
|  | **…** | **…** | **…** | **…** |
| Lowest scores | **encourage** | **hug** | humiliate | **hate** |
|  | **kick** | **hate** | **adore** | hug |
|  | kick | humiliate | **insult** | **hug** |

**Supporting Table S2.**

Social interactions sorted by their factor scores for the autism and control groups. The interactions shown in **bold** represent the agent role. Only the 3 interactions at each of the two extremes of each factor are shown; the factor scores for the intervening 10 interactions are similar to each other.

**Supporting References**

1. Just MA, Cherkassky VL, Aryal S, Mitchell TM (2010) A neurosemantic theory of concrete noun representation based on the underlying brain codes. *PLoS One* 5: e8622.
2. Tzourio-Mazoyer N, Landeau B, Papathanassiou D, Crivello F, Etard O, et al. (2002) Automated anatomical labeling of activations in SPM using a macroscopic anatomical parcellation of the MNI MRI single-subject brain. *NeuroImage* 15: 273-289.
3. Mitchell TM (1997) *Machine Learning*. New York: McGraw Hill.
4. Wang R, Benner T, Sorensen AG, Wedeen VJ (2007) Diffusion Toolkit: A software package for diffusion imaging data processing and tractography. *Proc. Intl. Soc. Mag. Reson. Med* 15: 3720.
5. Hess CP, Mukherjee P, Han ET, Xu D, Vigneron, DB (2006) Q-ball reconstruction of multimodal fiber orientations using the spherical harmonic basis. *Magn Reson Med* 56: 104-117.
6. Wakana S, Caprihan A, Panzenboeck MM, Fallon JH, Perry M, et al. (2007) Reproducibility of quantitative tractography methods applied to cerebral white matter. *NeuroImage* 36: 630-64
